# Fragment-Based Drug Discovery by NMR. Where Are the Successes and Where can It Be Improved?

**DOI:** 10.3389/fmolb.2022.834453

**Published:** 2022-02-18

**Authors:** Luca G. Mureddu, Geerten W. Vuister

**Affiliations:** Leicester Institute of Structural and Chemical Biology, Department of Molecular and Cell Biology, University of Leicester, Leicester, United Kingdom

**Keywords:** Fragments Based Drug Discovery, NMR-FBDD, Venetoclax, BCL-2, BACE-1, MCL-1

## Abstract

Over the last century, the definitions of pharmaceutical drug and drug discovery have changed considerably. Evolving from an almost exclusively serendipitous approach, drug discovery nowadays involves several distinct, yet sometimes interconnected stages aimed at obtaining molecules able to interact with a defined biomolecular target, and triggering a suitable biological response. At each of the stages, a wide range of techniques are typically employed to obtain the results required to move the project into the next stage. High Throughput Screening (HTS) and Fragment Based Drug Design (FBDD) are the two main approaches used to identify drug-like candidates in the early stages of drug discovery. Nuclear Magnetic Resonance (NMR) spectroscopy has many applications in FBDD and is used extensively in industry as well as in academia. In this manuscript, we discuss the paths of both successful and unsuccessful molecules where NMR had a crucial part in their development. We specifically focus on the techniques used and describe strengths and weaknesses of each stage by examining several case studies. More precisely, we examine the development history from the primary screening to the final lead optimisation of AZD3839 interacting with BACE-1, ABT-199 interacting with BCL_2/XL_ and S64315 interacting with MCL-1. Based on these studies, we derive observations and conclusions regarding the FBDD process by NMR and discuss its potential improvements.

## Introduction

Fragment Based Drug Discovery, or FBDD for short, is nowadays a well-established and common approach adopted by many pharmaceutical companies and academic groups (Erlanson et al., 2016). The rationale behind FBDD has been extensively reviewed and entire books have been written, including some with references to clinical candidates (Klon, 2015; Rees, 2016). The general concept of FBDD is straightforward; it starts with the generation of libraries of small molecules called “fragments.” The size of these libraries varies from a few hundred to thousands of molecules (for industrial cases) (Singh et al., 2018), usually prepared following the so-called “rule of three,” i.e. a molecular weight smaller than 300Da, lipophilicity as expressed by LogP smaller than 3, and a maximum number of hydrogen bond donors and acceptors less than 3 (Congreve et al., 2003). For efficiency reasons the binding against a target of interest is usually evaluated for each of these molecules in so-called “mixtures” of 5–10 compounds in a single experiment (Mercier and Powers, 2005). The strategy of using small molecules instead of large entities allows for a more efficient exploration of the chemical space, defined as the ensemble of all possible molecular conformations presenting drug-like properties, and estimated to be a staggering ∼10^60^ molecules (Reymond et al., 2010). The FBDD approach provides for a great chemical variety to probe this chemical space and has many other benefits, such as cost and time reduction in the data analysis.

Using fragments as a starting point in the early stages of drug discovery has been demonstrated to be a viable approach for producing compounds that are specifically tailored to their targets (Hughes et al., 2011). This approach also increases the novelty of standard drugs and enables the path of chemical optimisation to be monitored, for example by restricting the lipophilicity issue observed in lead molecules obtained by high-throughput screening (HTS) (Author Anonyms, 2007; Erlanson et al., 2016). However, since the fragments are much smaller compared to traditional lead-like molecules, their binding affinity to a target of interest is nearly always low (μM to mM). Therefore, it requires a technique, such as NMR, that is capable of detecting these weak interactions (Ortega-Roldan et al., 2009; Vinogradova and Qin, 2012).

In this manuscript, we focus exclusively on the relevance and impact of NMR spectroscopy on the generation of new clinical drugs. Through a literature review, we establish the impact of NMR in FBDD. We performed an in-depth assessment of three case studies that establish the impact of various NMR techniques on different stages of drug development. We establish patterns in the drug-development optimization process and formulate proposals for its improvement.

## Results

**FIGURE 1:**
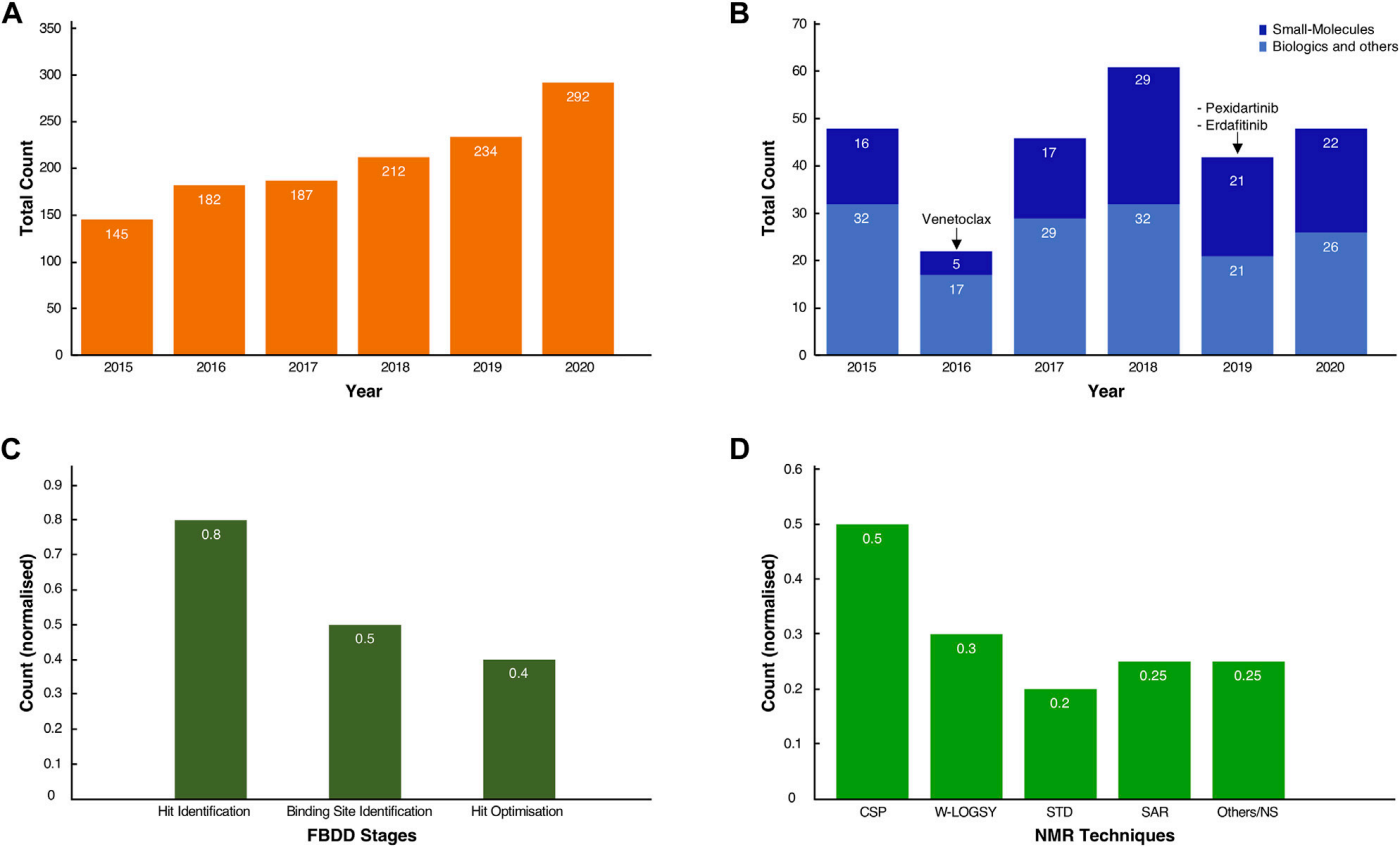
Usage of FBDD methods in the development of new molecules. **(A)** Total count of journal articles from Jan-2015 to Dec-2020 retrieved by querying “NMR and Fragment-based Drug discovery” in the PubMed Central database (see Supplementary Materials for the conditional query script). **(B)** Total count of FDA-approved New Molecular Entities (NMEs) and original biologics for the same time range. In dark blue, the small molecule NMEs, including the three known drugs whose fragment origins were derived from NMR studies. In light blue, the approved biologics, such as antibodies and oligonucleotides, and other chemicals entities such as diagnostics, combinations of old drugs, natural products. **(C)** Normalised scores of the occurrences of NMR spectroscopy as a technique in the discovery and development of molecules across the inspected cases. **(D)** Normalised scores for the total count of the various NMR techniques used throughout the drug discovery process of the inspected cases. Note that for both **(C,D)**, some compounds have been through multiple stages of drug discovery by NMR, so fractions sum to values >1.0.

To appreciate the influence on NMR in the current developments, we queried the PubMed database with the “fragment-based drug discovery” and “NMR” keywords. It revealed a steady and significant increase in the number of publications, growing from 145 in 2015 to 292 in 2020, for a total of 1,200 journal publications over this 6-year period (Figure 1A). A further search through the FDA database revealed, over the same time span, that several drugs were unmistakably obtained from fragments (Figure 1B) (Deeks, 2016; The ASCO Post, 2019). Excluding biologics (antibodies, oligonucleotides etc.) and other chemical compounds (diagnostics, combination of old drugs etc.), original small molecules make up a significant proportion, typically ∼40% or more, of the total FDA approvals in any given year (Figure 1B).

From the FDA database analysis, it appeared that only Venetoclax was developed with the help of at least one NMR technique in the early stages of the discovery. Whilst this may seem to be a low number relative to the total number of FDA approvals, this finding is likely explained by the fact that not every breakthrough in the drug development process obtained by NMR will have been shared in the public domain. Furthermore, despite our exhaustive literature search (*vide infra*), the origin of new molecular entities remained often obscure or very difficult to trace.

The true impact of NMR techniques in the development of original lead-molecules appeared clearer from the inspection of the origins of the clinical compounds. We performed an in-depth analysis of all FBDD-derived molecules that are or have been clinical candidates at any time in the past, up to December 2020, and for which relevant information was publicly accessible through journal articles, tables or from Dan Erlanson’s “Practical Fragments” blog (Erlanson, 2018). Our analysis showed that NMR is used in all three stages of the FBDD process (Figure 1C). For sixteen out of twenty clinical compounds (80%), we traced that NMR was used for identifying the initial binding fragments, the so called “hit identification” stage. For the subsequent FBDD stages, which include the “binding site identification” and “hit optimisation,” NMR was used less often, i.e. in 50 and 40% of the cases, respectively, (Figure 1C), with other techniques, such as X-ray crystallography, being preferred.

NMR-derived compounds were identified mostly by a number of ligand-detected, one-dimensional (1D) NMR techniques, such as Water-Ligand Observed via Gradient Spectroscopy (Water-LOGSY) (Antanasijevic et al., 2014), saturation transfer difference (STD) (Viegas et al., 2011; Meyer et al., 2004), or the so-called T_1ρ_-experiment (Hajduk et al., 1997). In contrast, target-based two-dimensional (2D) NMR techniques, such as the chemical shift perturbation (CSP) experiment (Mureddu and Vuister, 2019), were used for the hit and/or binding site validation. Lastly, the so-called SAR (structure activity relationship) by NMR method (Fesik et al., 1997; Shuker et al., 1996), which employs mostly NOE-related techniques and multi-dimensional NMR experiments, was mainly used for hit-growing and linking guidance during the optimisation stage of the FBDD process (Figure 1D) (Petros et al., 2006) (see also Supplementary Table S1).

The 1D ligand-detected techniques are considered a gold standard in NMR screening, as these do not require expensive protein labelling and therefore can be used against a broad range of molecular targets (Ciulli and Abell, 2007). Furthermore, the various expression systems of the target, e.g. bacteria, insects or human-derived cells, and other common limitations, such as molecular weights, are not of critical importance for these 1D techniques (Campos-Olivas, 2011). Moreover, the inherent versatility of NMR has also allowed the detection of the binding activity of small-molecules to receptors in their native environments and in in real-time, a strategy called *in-cell* NMR (Primikyri et al., 2018). Many common NMR techniques have been used in or adapted for *in-cell* NMR (Kang, 2019). However, STD and Tr-NOESY techniques (Mari et al., 2010) have been successfully employed without the limiting step of isotopic labelling (Primikyri et al., 2018).

In addition, 1D ligand-detected techniques can also be utilised in difficult cases where expression and/or purification of the target macromolecule is a limiting factor and only nanomolar concentrations can be obtained. Most importantly, the richness of information acquired in a small amount of time (i.e. minutes per sample) allows the analysis to be performed in a high-throughput fashion (Dalvit et al., 2002). However, 1D ligand-detected experiments are not suitable for detecting the binding sites on the target, and higher dimensionality NMR techniques, including chemical-shift perturbation mapping (Williamson, 2013; Mureddu and Vuister, 2019), are often required. The latter enables the monitoring of target residues that are most likely to be interacting with the fragments, providing validation of binding as well as guidance on the next stage of development (Williamson, 2013).

Fragment optimisation is best achieved where a high-resolution 3D molecular structure of the target is available. While there are several techniques capable of resolving molecular structures, the simplicity and the rapid throughput associated with X-ray crystallography, it makes this the preferred method whenever possible (Erlanson, 2015). However, often targets of interest cannot always be exhaustively assessed by X-ray crystallography. For example, complexes displaying a highly flexible mode of interaction with the target molecule are best inspected by NMR (Teague, 2003; Leone et al., 2010), as crystal packing forces preclude the molecular adaptation required for complex formation. Moreover, the crystal lattice also might not allow the ligand to permeate through to the binding pockets (Zheng et al., 2015). In contrast, the NMR technique can provide unambiguous information on the various orientations of the ligand with respect to the target, referred to as poses. These poses can be combined with computational methods for designing drug-like compounds with improved binding and pharmacological properties. Interestingly, the emergence of Artificial Intelligence (AI)-driven structure elucidation, such as AlphaFold-2 (Jumper et al., 2021) and RoseTTA fold (Baek et al., 2021) provides an additional avenue for the fragment-optimization stage. A description of the most recurrent NMR techniques to elucidate structure information is available in the supplementary materials (Supplementary Table S1).

In subsequent sections of this manuscript, we present three case studies that employed a variety of NMR techniques and therefore can be considered templates of NMR-FBDD. The first case study explores the development of a compound denoted as AZD-3839. It originated from fragments identified by a ligand-detected primary screening using the Water-LOGSY technique. The second case study examines the history of the FDA-approved drug ABT-199, commercially called Venotoclax, which was also derived by FBDD. The development of this drug relied on a variety of target-detected NMR methods. Finally, the third case study analyses a compound known as S64315, a recent FDA-approved drug, which illustrates the role of NMR in FBDD through a combination of ligand-detected and target-based NMR techniques.

### Case Study 1: AZD3839 and BACE-1

β-Site Amyloid precursor protein Cleaving Enzyme-1 (BACE-1) was identified over 20 years ago as a key component in Alzheimer disease (AD) pathogenesis (Vassar et al., 1999; Venugopal et al., 2008). BACE-1 is responsible for the initial cleavage of the amyloid precursor protein into smaller amyloid β-peptides (Aβ), whose accumulation in brain cells is believed to be one of the underlying causes of AD progression (Vassar et al., 2009). Not surprisingly, BACE-1 is a therapeutic target and a number of academic groups and pharmaceutical companies have placed considerable efforts into the research and development of new inhibitors in the hope of limiting or blocking the formation of Aβ (Ghosh et al., 2012; Goyal et al., 2014; Erlanson and Janke, 2016; Hung and Fu, 2017).

BACE-1 is characterised by an internal groove created by two lobes (S1 and S2), modulated by a loop (“flap”) which reveals the aspartyl catalytic site. The flap is highly dynamic, and the presence of an inhibitor can determine whether the macromolecule is in the “open” or “closed” state, thus modulating access to the catalytic pocket. The identification of two crucial aspartic acid residues, i.e. Asp32 and Asp228, has for many years driven the drug development process and optimisation of fragments (Erlanson and Janke, 2016). An exhaustive list of early fragments and their respective primary screening techniques is given by Erlanson and Jahnke in their “Lessons and Outlook” book (Erlanson and Janke, 2016).

A great example of the history of a complete development of a BACE-1 inhibitor is given by the compound AZD-3839, where the initial fragment was identified from 1D NMR studies. According to Geschwindner *et al.*(Geschwindner et al., 2007) at AstraZeneca the choice of NMR for this case provided a compromise between scalability of large fragment libraries with sufficient data-output on the one hand, and ensuring a robust method for detecting very weak binding at low ligand concentration on the other hand. By eliminating non-specific binders, the process also had the advantage of reducing false positives from the analysis.

**FIGURE 2:**
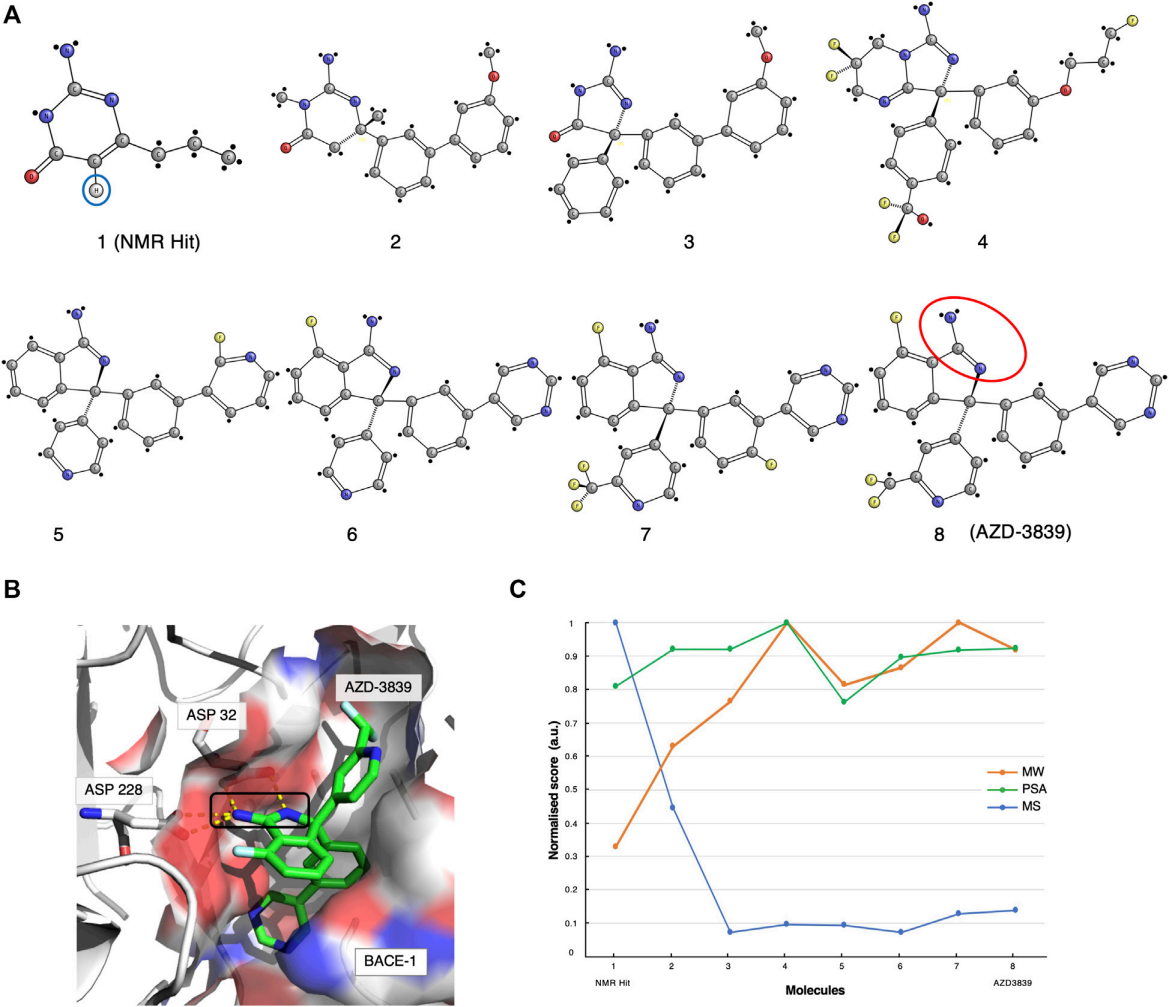
The AZD-3839 case-study. **(A)** The optimisation pathway: from NMR hits to AZD-3839. Compound-**1** represents the hit initially identified from the Water-LOGSY NMR study. The blue circle highlights the crucial isocytosine aromatic proton. Compounds were optimised through a series of crystallography-based methods to yield the final compound-**8** (AZD-3839) (Geschwindner et al., 2007; Jeppsson et al., 2012), yet preserving the original amidine motif (red circle) already present in compound-**1**. **(B)** Molecular structure representation of BACE-1 (PDB code: 4B05) and the main interaction between the catalytic groove (Asp32 and Asp228) and the amidine group of AZD-3839, first observed in the NMR-discovered hit (black rectangle). **(C)** Molecular similarity, as expressed by the Tanimoto coefficient (MS, Blue), normalised molecular weight (MW, orange) and polar surface area (PSA, green) scores for the eight compounds on the development path of AZD-3839.

The original screen using the Water-LOGSY 1D NMR technique was conducted on a 2000-compound library with four fragments per mixture, yielding a relatively low hit rate of 0.5%. Compound-1 (Figures 1, 2A, ) was identified as a binding hit. The hallmark intensity “sign-flip” of the Water-LOGSY signals resulting from this compound was clearly identifiable in the NMR spectra, suggesting binding to BACE-1 (Geschwindner et al., 2007). Crucially, as a control, the authors performed a competition experiment in the presence of a stronger known binder, showing a noticeable intensity reduction (signal becomes more negative) only for the singlet peak from the isocytosine aromatic H^5^ proton at around 5.65 ppm (Figure 2A, compound 1, blue circle). This validation assay reduced potential false positives by identifying fragments that displayed weak binding Water-LOGSY responses but did not show any changes upon the addition of the competitor. Compound-1 was eventually selected for further optimisation steps.

Meanwhile, through parallel crystallographic studies performed by Astex Therapeutics (Murray et al., 2007) an optimised compound that preserved the original amidine motif was developed. The amidine motif was confirmed to be responsible for the strong interaction to the catalytic aspartates (Figure 2B). Subsequently, through a series of substitutions on the crucial scaffold, the molecule was morphed into the isoindole present in the final compound. Furthermore, the introduction of fluoro atoms improved the permeability of the molecule and the brain exposure by “shielding” the reactive amidine. Lastly, additional molecular interaction surface on the molecule, needed for the interaction of the molecule with the adjacent S3 and the flap (Edwards et al., 2007) (Figure 2B), was created by extension with additional aromatic moieties.

The various steps of this hit optimisation process clearly show how the initial NMR-derived fragment has undergone a series of dramatic changes. The magnitude of these changes can be assessed from the similarities of the molecular characteristics for each component, as given by its molecular weight (MW), polar surface area (PSA) and by the Tanimoto coefficient (Bajusz et al., 2015) which measures molecular similarity (MS, Figure 2C). A prominent drop of the Tanimoto coefficient is observed from the first NMR-detected compound-1 to compound-2 and further for compound-3. However, from compound-4 to the final AZD-3839 molecule, a much smaller variation in the score is observed. A different trend was observed for the molecular weight of successive compounds which showed a constant increment up to compound-4, followed by only minor changes towards the final AZD-3839. Interestingly, the final compound was characterised by a ∼10% smaller molecular weight compared to its predecessor yet attained a slightly increased PSA value (Figure 2C).

AZD-3839 appeared to be a very promising drug candidate and underwent clinical phase-1 trials. Unfortunately, it was stopped from patient administration, probably due to its high affinity to the hERG ion channel and resulting related side-effects (Berg et al., 2012). Nevertheless, this case demonstrated that NMR was crucial for determining the first hit from the primary screening containing the essential amidine-fragment. This motif proved to be of a critical importance in the interactions to BACE-1, and as a result it was preserved through the long path of further chemical optimisations that resulted in the final AZD-3839 compound.

### Case Study 2: ABT-199 (Venetoclax) and BCL-2_/XL_

The second case study concerns the analysis of ABT-199, commercially referred to as Venetoclax, that obtained FDA-approval as BCL-2 inhibitor in 2016. This drug was selected for our studies for two reasons: firstly, the large impact of NMR throughout its development pathway and secondly because the Abbott NMR group, who initiated the studies on ABT-199, have pioneered the so-called “SAR by NMR” method that also underpinned its development (Shuker et al., 1996; Fesik et al., 1997) α.

**FIGURE 3:**
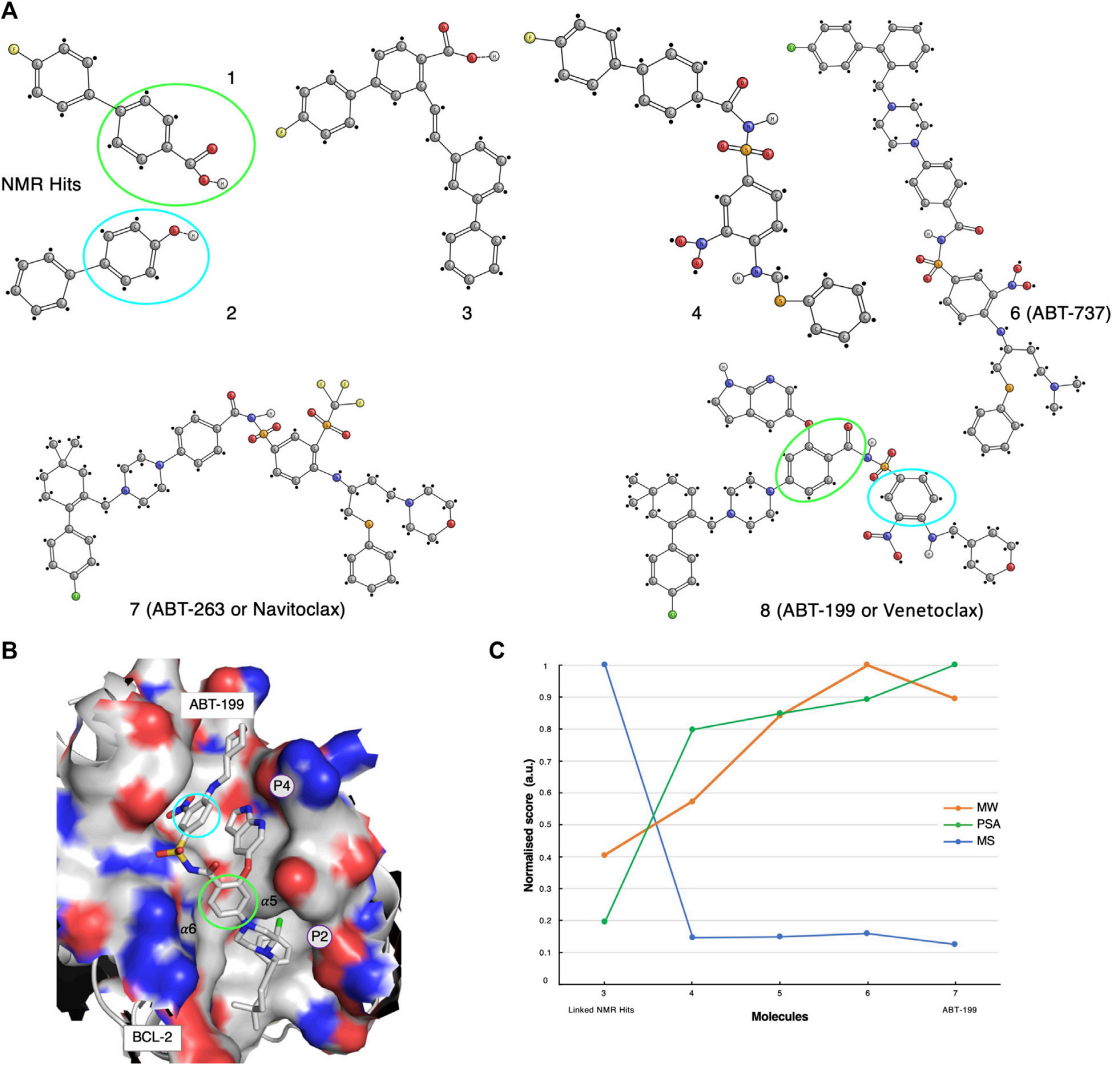
The ABT-199 case-study. **(A)** Optimisation pathway: from NMR hits to ABT-199. The aromatic moieties of the NMR-determined hits (green and cyan circles) were originally identified as interacting with BCL-XL active sites (Petros et al., 2006). Compounds 1, 2, 3, 4 were identified and optimised through NMR methodologies, whereas the latest ABT compounds optimisations benefited from X-ray crystallography techniques (Petros et al., 2006; Petros et al., 2010; Souers et al., 2013). **(B)** Molecular structure representation of BCL-2 in complex with Venetoclax (PDB code: 6O0L). Green and cyan circles indicated the aromatic motifs originally identified through the NMR primary screening. **(C)** Molecular similarity, as expressed by the Tanimoto coefficient (MS, Blue), normalised molecular weight (MW, orange) and polar surface area (PSA, green) scores for compounds 3-7 on the development path of ABT-199.

ABT-199 is an inhibitor of the anti-apoptotic proteins BCL-2, BCL-xL, and BCL-w (Souers et al., 2013). These proteins play a pivotal role in cell survival; not surprisingly, they are over-expressed in many cancers and they are directly linked to initiation, progression and therapy resistance occurrences (Choudhary et al., 2015). The BCL members are α helical proteins; two of these, BCL-2 and BCL-xL, share four domains, BH4 (α1), BH3 (α2), BH1 (partially α4), and BH2 (partially α6 and α7) plus the transmembrane, TM, motif. The two central hydrophobic helices (α5 and α6) together with the amphipathic α1-4 and α7 together form an elongated hydrophobic groove in the so-called BH1, BH2, BH3 regions (Kelekar and Thompson, 1998) (Figure 3B). The BH3 region, in particular, is responsible for the interaction with other proapoptotic proteins such as BAK and BAX, rendering it a druggable site of interest (Letai et al., 2002).

The early inhibitor-discovery process was started by screening a large library of small molecules using 2D target-detected approaches, which led to the identification of several molecular candidates (Figure 3A, compounds 1 and 2). The hypothetical binding mechanism was elucidated through ^15^N-HSQC chemical shift perturbations (CSP) experiments (Petros et al., 2006). From the CSP results, it was possible to derive that the fluoro-biaryl acid region of compound-1 interacted with the BCL-xL hydrophobic groove. In fact, a series of shifts were observed for the peaks assigned to BCL-xL residues Gly94, Gly138, and Gly196, located in this groove (Petros et al., 2006). However, the study of the complex of BCL-xL with its binding partner BAK suggested the existence of an additional binding interface. Therefore, a second NMR screening was carried-out in the presence of a large excess of compound-1, with the aim of saturating the first site of interaction and while screening for potential hits to the second interface (Petros et al., 2006). Compound-2 (Figure 3A) was identified and eventually chosen to be used for chemical linkage to compound-1, in-line with the “SAR by NMR” approach (Petros et al., 2006). Multiple linkers, derived on the basis of various poses of compound-1 in the BH3 binding groove, were explored in order to improve the overall potency of the resulting molecule. Finally, a ∼200-fold improvement in binding affinity was established for compound-3 when compared to the original compound-1 (Figure 3A).

The first molecular model of the complex of BCL-xL with compound-3 was then derived on the basis of nine intermolecular NOEs (Petros et al., 2006). Although these NOEs were indicative of an interaction with both binding interfaces, it was concluded that compound-3 did not adopt optimal or ideal conformations. Consequently, new linkers and a new set of chemical reactions for connecting the fragments were explored.

Compound-4 was eventually synthesised and structurally evaluated on the basis of protein-ligand NOEs extracted from 3D^13^C-edited and ^12^C-filtered NOESY spectra, that allowed the molecule to be docked in the BH3 groove (Petros et al., 2006). Compound-4 was further optimised for those parts that were solvent-exposed. These parts of the molecule were replaced with polar substituents, including a 2-dimethylaminoethyl group in the linker. In addition, the insertion of a new piperazine ring resulted in compound-6, also referred to as ABT-737 (Figure 3A). ABT-737 displayed biological activity in the presence of human serum. A crystal structure at 2.2 Å resolution of BCL in complex with ABT-737, solved after the original NMR-determined binding pose of compound-4, validated the latter (Lee et al., 2007). The structure showed that ABT-737 interacted with the two binding interfaces formed by the hydrophobic pockets, P4 and P2, of BCL-2 and BCL-XL; including two hydrogen bonds from the thiophenyl and the 1-chloro-4-(4,4-dimethylcyclohex-1-enyl)benzene moieties to residues Gly138 and Glu96, respectively.

An *in-vivo* analysis suggested that synergetic therapy was required to inhibit the anti-apoptotic activity of the BCL family, while simultaneously promoting the activity of the pro-apoptotic proteins (BAX and BAK) (Oltersdorf et al., 2005). Therefore, the Abbott group also developed the ABT-263 molecule. After an initial positive assessment on multiple cellular lines, where ABT-263 reported stronger inhibitory actions, presumably by targeting both BCL-xL and BCL-2, advanced clinical studies unfortunately revealed major physiological pitfalls such as thrombocytopenia (Vandenberg and Cory, 2013).

Meanwhile, the project progressed to the final compound ABT-199 (Venetoclax) from ABT-263 through a series of substitutions (Vandenberg and Cory, 2013). In addition, new 3D crystal structures of BCL-2 in complex with various ligands were made publicly available (PDB codes listed in Supplementary Material and Methods) (Souers et al., 2013; Lee et al., 2007; Birkinshaw et al., 2019). The ABT-199 molecule (Figure 3A, compound-8) incorporated several crucial modifications compared to its ABT-263 (Figure 3A, compound-7) predecessor; a pivotal H-bond to Asp103 (corresponding to Glu96 in BCL-xL) was identified, thus providing an increased affinity to both the BCL-2 and BCL-xL P4 pockets (Souers et al., 2013) (Figure 3B).

We performed a molecular fingerprint analysis for all available molecules in the development process. However, as the initial NMR-detected fragments underwent a linkage step, the molecular similarities were assessed with respect to compound-3 (Figure 3A). Similarly to the AZD-3839 case study, a drastic drop in the Tanimoto coefficient was observed from compound-3 to the following optimised forms (Figure 3C). Interestingly, ABT-199 showed a reduced molecular weight and increased polar surface area compared with its predecessor compounds yet keeping an overall structural similarity relative to compound 4.

The ∼20-years development history of the ABT-199 compound revealed a multitude of challenges, including the initial failure in obtaining crystals of complexes with the first leads and various other *in-vivo* difficulties, which were not predictable from a structural point of view. However, our analysis shows that the development of Venetoclax most likely would not have been achieved without the crucial data obtained from multiple NMR techniques at the various stages of the ABT-199 drug-development process.

### Case Study 3: S64315/MIK665 and MCL-1

**FIGURE 4:**
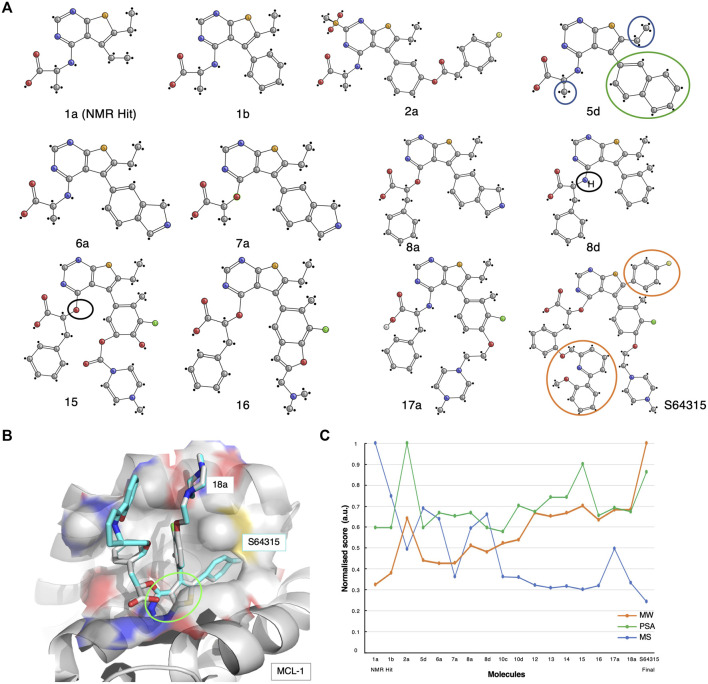
The S64315 case-study. **(A)** Optimisation pathway: from NMR hits to S64315. All compound nomenclatures are identical to those used in the original manuscript (Szlávik et al., 2019) for an easier comparability. Compound **1a** represents the initially identified thienopyrimidine core by ligand-detected 1D NMR techniques. The green and blue circles for compound **5d** highlight the chemical groups that gave rise to crucial NOEs that suggested the initial molecule binding poses (Szlávik et al., 2019). **(B)** Molecular structure representation of a model of MCL-1 in complex with compound **18a** (PDB code: 6QYO). The green ellipse highlights the original thienopyrimidine motif first identified by an 1D-NMR screening experiment (Szlávik et al., 2019). **(C)** Molecular similarity, as expressed by the Tanimoto coefficient (MS, Blue), normalised molecular weight (MW, orange) and polar surface area (PSA, green) scores for the twelve compounds on the development path of S64315 AZD-3839.

The third case study presents an overview of the most crucial optimisation steps in the development of the molecule S64315, also known as MIK665 (Szlávik et al., 2019) (Figure 4A). S64315 is one of the most recent inhibitors currently being tested in clinical trials for targeting the BCL anti-apoptotic family, MCL-1 (Maragno et al., 2019). A series of studies indicated that MCL-1 is over-expressed in many cancer types (multiple myeloma, lymphomas, leukaemia) and therefore it is widely recognised as a druggable target (Albershardt et al., 2011). MCL-1 shares the highly conserved BH3 binding groove with other members of the family such as BCL-xL and BCL-2 (*vide infra*). This groove is essential for interacting and sequestering the pro-apoptotic proteins resulting in an increased cell survival.

The development of specific inhibitors for MCL-1 which target the BH3 groove has proven to be challenging (S. Soderquist and Eastman, 2016). Researchers at Vernalis, together with collaborators, engaged in extensive efforts in their studies of this complex. These studies maximised the potency of an initial hit that was obtained from a ligand-detected NMR screening, and resulted in the most promising MCL-1 inhibitor to date, S64315/MIK665 (Szlávik et al., 2019). However, several difficulties had to be overcome during its development, such as the lack of 3D atomic structures resulting from the poor expression and purification of MCL-1 in human-cell lines (Szlávik et al., 2019). Despite this, the protein availability proved adequate for the initial NMR-based screening.

A thousand initial compounds, pooled in groups of eight, were screened using various 1D NMR techniques, such as STD, Water-LOGSY and relaxation experiments (cf. Supplementary Table S1), to reveal several potential binding hits. Due to low signal-to-noise ratios of the screening experiments because of limited sample availability, hits were further validated using 2D NMR ^15^N-HSQC titrations. In addition, to overcome the lack of a detailed 3D molecular structure, a new approach for determining ligand poses and guiding the drug development optimisation process was developed. This approach, referred to by the researchers as the NMR-guided model (NGM), employs 3D NMR methodology, i.e., X-filtered NOESYs (^13^C-edited,^13^C,^15^N-filtered), to identify crucial NOEs between ligands and the target. The information resulting from these NMR studies was combined with high-throughput computational docking studies, allowing for a more accurate classification of binding poses. From the NMR results, multiple compounds with various chemical functionalities were explored, of which a class of compounds comprising a thienopyrimidine group were believed to be the most promising. Particularly, NMR-derived compound-1a was used as the initial fragment towards the development of the S64315 drug (Figure 4A). Following a series of substitutions for the compound’s ethyl group, multiple variants were tested on BCL-2, BCL-xL and MCL-1. Some of the newly synthesised molecules showed comparable affinity toward all three targets (Szlávik et al., 2019). Using the ^15^N-HSQC technique, it was possible to estimate K_d_ values for most of these, which ultimately allowed the selection of compound**-**5days as the highest affinity binder for MCL-1.

**FIGURE 5:**
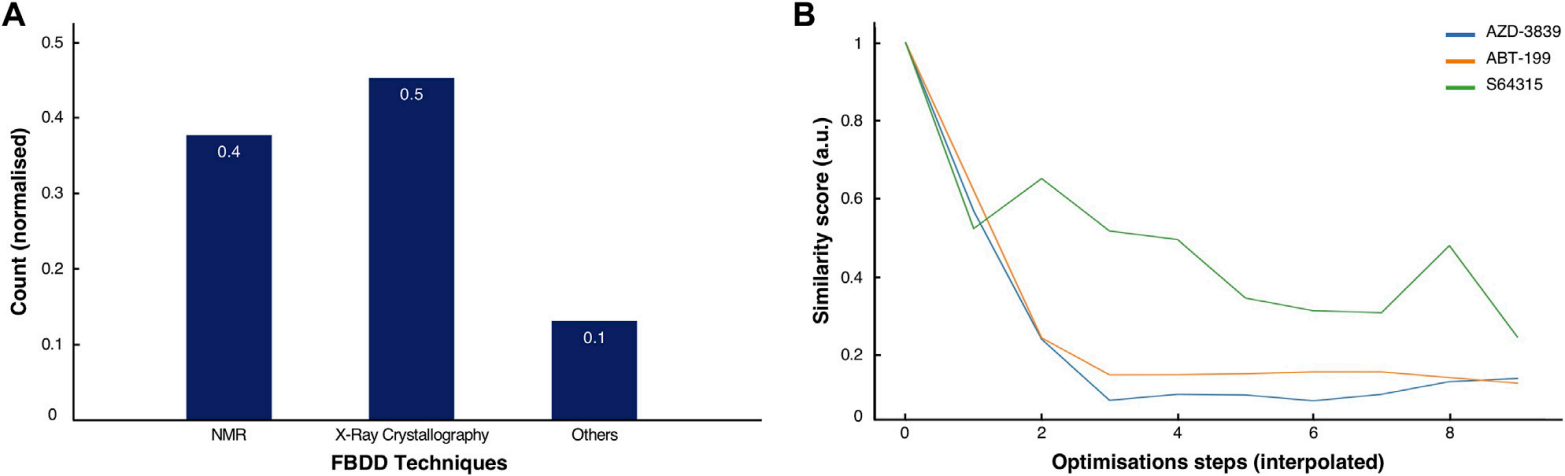
Comparison of methods and history of clinical drugs. **(A)** Normalised score of the predominant methodologies used for the discovery and development of the 53 clinical drugs inspected in this study. **(B)** Normalised similarity scores for the ABT-199 (orange), the S64315 (green) and the AZD-3839 (blue) fragment-to-drug developments pathways with interpolated optimisations steps.

The NOEs derived from the analysis of the compound-5days/MCL-1 complex indicated several potential contacts. In particular, contacts between the naphthyl ring (Figures 4A, 5D, green circle) and the MCL-1 side chains of Ala227, Met231, Val249, Val253, and Thr266 were observed as well as between a methyl group (Figures 4A, 5D, blue circles) and the side chains of Met231, Val249, Val253, Leu267 (Szlávik et al., 2019). To further investigate the BH3 binding region’s molecular flexibility, the researchers assessed various possible docking poses using multiple structural ensembles. This approach enabled a better estimation of the possible allowed geometries that were consistent with the experimental NOE information. Ultimately, the preferred molecular orientation consisted of the carboxylic acid pointing toward the solvent region, and the naphthyl group toward the S2 pocket. Different conformations and variations of the ligand molecule interacting with the hydrophobic groove were also assessed. This was achieved by modifying the core of the original compounds by inserting various aliphatic substituents and testing the different rotational properties of the resulting aryl ethers and anilines (Figure 4A, 8days-15, black circles).

At a much later stage, crystallographic structures of the MCL-1 complex and some variants became available (PDB codes listed in Supplementary Material and Methods), allowing for more detailed studies of several fragments and their binding modes.

Multiple optimisation steps were carried out, eventually leading to S64315 (Figure 4A, compound-8a). This final compound presented new crucial ortho-substituents, such as the fluorobenzene and methoxyphenyl-pyridine group (Figure 4A, orange circles), which were responsible for the increased selectivity for MCL-1 compared to its precursor. Here, we have generated a model of MCL-1 in complex with compound-18a (Szlávik et al., 2019) using the MCL-1 crystal structure (PDB code 6QYO) by manually overlaying the S64315 molecule onto compound-18a (Figure 4B). The model shows the thienopyrimidine motif, already observed in the original NMR-derived compound-1a, deeply buried in the hydrophobic groove of the BH3 binding domain.

As for the previous case-studies, we inspected the molecular characteristics using the normalised MW and PSG scores and the Tanimoto score for all available compounds (Figure 4C). In line with the observations for both ABT-199 and AZD-3839, albeit somewhat less prominent, the result of this analysis again shows the characteristic initial decrease in molecular similarity from the first fragment to the following variants, indicating the significant changes during the initial steps of development. Interestingly, several compounds mid-way through the development (i.e., compounds 5 days, 8 days) showed a higher Tanimoto coefficient compared to the initially optimised fragments, suggesting a more careful optimisation process rather than a revolutionary approach to the first NMR-derived hit. Starting from compound-10 only smaller changes occur together with increased PSA scores. Surprisingly, the final compound appeared to differ the most from its direct precursors. This compound also showed an increased MW and a reduced PSA score compared to its previous three variants.

The search for an MCL-1 inhibitor started several years ago from the identification of a first hit obtained through primary screening by NMR. The process illustrates the huge amount of work required to bring an initial hit to a final lead drug candidate, which included the efforts of multiple academic and industrial laboratories. The failure of crystallisation trials during the early stages of the project, plus the inherent flexibility of the MCL-1 BH3 binding groove, made NMR spectroscopy uniquely capable of driving the project forward. The S64315 compound is currently under evaluation in the clinical phase-1 trials, which provides hope for patients affected by a variety of cancer types.

## Discussion and Conclusion

We explored the development histories of the AZD-3839, ABT-199 and S64315 compounds (Jeppsson et al., 2012; Souers et al., 2013; Szlávik et al., 2019), from the primary screening to the final lead optimisation, focusing on their target interactions and the rationale behind their optimisations. These cases highlighted the underlying role of NMR techniques during all drug discovery phases and their impact throughout each stage.

With multiple compounds in clinical phases, NMR has demonstrated a key role in the process of fragment-based drug discovery (Singh et al., 2018) (Figure 5A). In 2016, ABT-199, commonly known as Venetoclax, was the first confirmed FDA-approved drug derived largely by NMR-FBDD (Erlanson, 2012; Souers et al., 2013; Hubbard, 2016). The development of other fragment-derived drugs, e.g., Vemurafenib (approved in 2011) (Flaherty et al., 2011), Erdafitinib (approved in 2019) (Perera et al., 2017; The ASCO Post, 2019), Pexidartinib (approved in 2020) (Benner et al., 2020) and Sotorasib (approved in 2021) (Ostrem et al., 2013), were driven by both NMR, X-ray crystallography and other techniques.

Interestingly, Vemurafenib, and Pexidartinib, both developed by Plexxikon, the phase-III drug Capivasertib (Addie et al., 2013) developed by Astra-Zeneca, and many other clinical candidate molecules, all include the identical 7-azaindole fragment (Irie and Sawa, 2018; Qhobosheane et al., 2020). This unique structural composition presents both crucial hydrogen-bond acceptor and donor groups making this small molecule able potentially to interact with over 90 different kinds of kinase active sites, which has been considered encapsulating the entire human Kinome (Irie and Sawa, 2018).

In spite of this distinctive case of a generally adaptable building block, over the years, different methodologies have been explicitly developed and proven crucial for enhancing the success rate in the drug discovery process (Pellecchia, 2010). The great flexibility and adaptability of NMR provides for qualitative and quantitative insights at each point of the drug development process (Campos-Olivas, 2011; Pellecchia, 2010). From the case studies, it emerged that NMR is predominately used in the primary screening, also known as “hit-identification” (Figure 1C). The detection of weak binders, defined as small-molecules presenting transient interactions to targets or unfavourably high dissociation rates, is paramount in the early stages of FBDD (Wang et al., 2017). The identification of such compounds represents a clear advantage as it shows its natural ability to bind its target and succeed among cocktails of other sub-molar molecules. The crucial chemical nature of these molecules, i.e., their “core”, is often used as a scaffold and preserved during their evolution to final drugs (Figures 3A, 4A).

However, the process hit-identification by NMR has also shown a number of drawbacks (Davis and Erlanson, 2013). For example, the usage of only a single ligand-detected 1D technique for identifying binding fragments may prove to give erroneous results (Davis and Erlanson, 2013). Hence, the recommendation is to use at least two NMR techniques, such as STD and Water-LOGSY (Davis and Erlanson, 2013) in parallel.

An inevitable consequence of using weakly binding fragments is the so-called “non-specific” binding event (Mercier et al., 2009). A simple strategy for alleviating this issue was employed in the development of AZD-3839. By recording competition experiments using a potent known, but clinically unsuitable, ligand for the same target non-specific binding molecules were identified and excluded from further development.

Furthermore, from the case studies it appeared that the full analytical power of 1D NMR spectroscopy was often not exploited. Instead, the NMR data appear to be used solely as a binary result, probably also due to a lack of proper computational- and data-analysis tools available at the time the research was conducted (Mureddu et al., 2020). The data obtained from Water-LOGSY and STD experiments offer further quantitative information (Meyer et al., 2004; Cala and Krimm, 2015). The SAR by Water-LOGSY, for example, suggests a scoring factor to identify the most exposed portion of the molecule. Assessing all data that can be derived from 1D and multi-dimensional NMR experiments can eventually provide insights into the ligand binding pose (Raingeval et al., 2019).

Upon validation of fragment hits, the next stage is usually the exploration of potential binding sites on the target. Chemical shift perturbation, or CSP, is so far the most popular NMR technique used for this task (Williamson, 2013; Mureddu and Vuister, 2019). CSP has been widely used as a standard for molecules that progressed into clinical phases. Nevertheless, a CSP analysis potentially might drive researchers in wrong directions, and final conclusions should not be based on this approach alone. Common errors observed in practice include subjective judgments or misinterpretation of shifting peaks, especially in crowded regions of the spectra, leading to overestimating of the CSP effects. Furthermore, in some instances, compounds have been shown to change the pH of the solution, resulting in false positive CSPs (Davis and Erlanson, 2013). By performing appropriate control experiments, such errors might hopefully be avoided.

NMR has often been associated with a requirement for daunting and time-consuming data analysis. There may be a multitude of other undescribed factors, but NMR’s lack of modern, more practical, quicker, and unbiased methods, alongside with automated data analysis routines has comparatively slowed the entire NMR-FBDD process, prompting a need for improvements in all these aspects (Mureddu et al., 2020). To this end, we have recently developed a versatile and flexible data-analysis program called AnalysisScreen (Mureddu et al., 2020), part of the CcpNmr Analysis package (Skinner et al., 2016), which presents dedicated provision and capabilities for the analysis of all forms of NMR data used in the NMR-FBDD process, including an integrated CSP analysis (Mureddu and Vuister, 2019).

An additional key step before the expensive and laborious optimisation processes of candidate compounds begins is a minute hit chemical assessment; for example, by employing Pan-Assay Interference Compounds (PAINS) protocols (Baell and Nissink, 2018). Applying these filters to hits or families of hits can help identify erroneous binders. PAINS-flagged molecules can exert photo-reactivity, redox-activity and other undesirable chemical phenomena, which can lead to non-specific or unwanted biological activities. Unfortunately, it is also wise not to rely solely on PAINS filters. A recent analysis showed that many PAINS-flagged molecules had been wrongly evaluated by the applied filters, either as false negatives or false positives (Capuzzi et al., 2017).

Ayotte *et al.* proposed the use of a NMR CPMG series to detect potential aggregation of compounds in mixtures (Ayotte et al., 2019), another potentially complicating effect. In addition, the authors also pointed out that aggregation can be solvent-dependent, and thus a minor adjustment of the sample composition might improve or worsen the outcomes of screening experiments (Ayotte et al., 2019).

The methodological and data-analysis improvements are fundamental to design the molecules from the early stages and onward. From the analysis of the three cases, it emerged that molecular optimisations are mainly guided by multiple manual moiety substitutions, followed by their chemical synthesis and re-evaluation. Although this might generate potential leads, a meticulous use of computational approaches could likely have accelerated this process further. Molecular docking studies aided the final lead generation of the ABT-199 compound; however, combining NMR and molecular docking can still introduce mistakes and whereas docking alone can also not be fully trusted (Chen, 2015). Even the newest scoring functions, implemented using artificial intelligence (AI), likely still present significant limitations (Gabel et al., 2014), as incomplete or erroneous classification of existing experimental data can compromise and bias their validity (Uçar et al., 2020).

Despite these setbacks, we firmly expect that newer chemo-informatics and AI algorithms, together with improved high-performance computing resources for rapid parallel in-silico drug screening using Molecular Dynamic (MD) simulations, will replace the current optimisation stages of the FBDD protocols. Several algorithms can already complement the experimental data validation (Bienstock, 2011). In addition to experimental methods, such as X-ray crystallography, NMR and electron microscopy, AI-driven structure elucidation by tools such as AlphaFold-2 (Jumper et al., 2021) and RoseTTA fold (Baek et al., 2021) can also provide the 3D molecular information required for the fragment-optimization stage. Ideally, the computational approaches for fragment optimization should simultaneously take the interaction, the experimental data such as derived by NMR, as well as biological aspects into consideration. For example, the robustness of a candidate compound or potential drug resistance could also be assessed early in the development process, thus integrating multiple fields of drug-discovery and pharmacology in a holistic way that scientists alone could not have attained (Dey and Caflisch, 2008).

By analysing the molecular characteristics of the various compounds, we have identified correlations in the development patterns among the three cases studied. By comparing the Tanimoto coefficients (Bajusz et al., 2015) for the three final compounds (Figures 2C, 3C, 4C), we speculate that expanding the molecule (via growing/linking methods) did not improve the binding affinity. This suggests that just covering the conformational molecular interaction space does not necessarily lead to higher affinity drugs. Figure 5B displays the normalised Tanimoto scores for the three different fragment-to-drug evolutions. Clearly, the development of AZD-3839 and ABT-199 display a highly similar pattern, which is dissimilar from S64315. An obvious conclusion could be that they simply differ in their optimisation protocols. However, it could equally suggest that the optimization of S64315 is not yet complete and multiple additional changes might occur before “converging” to the ultimate drug.

In conclusion, although only a few drugs approved by FDA have an NMR fragment-based trackable history that is easily accessible from public domain data, an analysis of relevant publications over the last 6 years shows an impressive number of journal articles reporting on discoveries of molecules in the pre-clinical stages in which NMR had a crucial role (Figure 1A). One of these molecules, Asciminib (Schoepfer et al., 2018), has only just recently (2021) been granted a FDA Breakthrough Therapy designation (Furnari et al., 2021), and it constitutes yet another marvellous example of the crucial role of NMR experiments in every stage of its development (Schoepfer et al., 2018).

In all, the overall methodological advances in the steps leading from initial hit to candidate-drug provide great hope that, compared to the time currently taken, new potent and selective drugs will soon become much more rapidly available.

## Data Availability

The datasets presented in this study can be found in online repositories. The names of the repository/repositories and accession number(s) can be found below: https://github.com/VuisterLab/scripts.git.

## Abbreviations

BACE, Beta-Site APP Cleaving Enzyme; BCL, B-Cell Leukaemia/Lymphoma; CPMG, Carr-Purcell-Meiboom-Gill; Cryo-EM, Cryogenic electron microscopy; CSP, Chemical Shift Perturbation; FDA, Food and Drug Administration; HSQC, Heteronuclear Single Quantum Coherence Spectroscopy; MBP, Maltose-Binding Protein; MCL, Myeloid Cell Leukaemia; MD, Molecular Dynamics; MW, Molecular Weight; NMR, Nuclear Magnetic Resonance; NME, New Molecular Entities; NOE, Nuclear Overhauser Effect; NOESY, Nuclear Overhauser Effect Spectroscopy; PAINS, Pan-Assay Interference Compounds; PSA, Polar Surface Area; SAR, Structure Activity Relationship; SPR, Surface Plasmon Resonance; STD, Saturation-Transfer Difference; W-LOGSY, Water-Ligand Observed via Gradient Spectroscopy.
