# Supplementary material for: Fragment-Based Drug Discovery by NMR. Where Are the Successes and Where can It Be Improved?
Source: Front Mol Biosci. 2022 Feb 18;9:834453. doi: 10.3389/fmolb.2022.834453 (PMC8895297; doi:10.3389/fmolb.2022.834453)
Supplement: Supplementary file 1 [file DataSheet1.pdf]

Fragment-based drug discovery by NMR. Where are the successes and where can it be improved?

*Luca G. Mureddu and Geerten W. Vuister**

Leicester Institute of Structural and Chemical Biology, Department of Molecular and Cell Biology, University of Leicester, United Kingdom

*Correspondence:

Corresponding Author: gv29@leicester.ac.uk

1.1. Supplementary Materials and Methods

1.2. Materials

FDA information for New Molecular Entities (NMEs) and original biologics were extracted from https://www.fda.gov/drugs/drug-approvals-and-databases/drug-trials-snapshots. For each entry, a literature search was conducted to determine whether small molecules (only) were derived from an FBDD-approach.

The list of molecules in various stages of clinical trials was reproduced from a number of web sources and published reviews[1–4]. Some web-based materials were extracted from a detailed analysis of the website Practical Fragments[5], where blog articles dating up to 31st December 2020 and a table as listed in 2015 and 2018 posts[6,7] provided the starting point for this study.

A literature review was conducted for each compound, filtering out only molecules in which NMR had been involved at some stage of the drug discovery process. Subsequently, the exact NMR technique used was noted wherever possible. Statistics were derived from publicly available

resources, including databases and web blogs, therefore, they could include errors, inaccuracies or be incomplete.

1.3. Methods

Fragments molecular structures were reproduced using our CcpNmr software ChemBuild[8]. OpenBabel or iBabel 3.6 was used to convert PDB and MOL2 to SMILES format[9,10]. Smiles were created in the canonical (xc) format; hydrogens and pH were excluded from the calculations of the various molecular properties. Molecular weights, polar surface areas and other properties were calculated using the online tools available at http://www.cheminfo.org.
A collection of scripts for analysing smiles and plotting molecular similarities were written in Python using the Pandas[11], Numpy[12], SciPy[13], Matplotlib[8] libraries. The Pybel[9] package was used for calculating the molecular fingerprints from SMILES and the Tanimoto coefficient[14]. Scripts and raw data are available in the Vuister Lab GitHub repository at https://github.com/VuisterLab/scripts.git.

PDB codes used:

- AZD-3839 and BACE-1: 4B05;
- BCL and ligands: 6O0L, 4LVT, 2YXJ;
- MCL1 and ligands: 6QXJ, 6QYK, 6QYL, 6QZ5, 6QZ7, 6QZ6, 6QZB, 6QYN, 6QZ8, 6QYP, 6QYO.

PubMed Central searching query (https://www.ncbi.nlm.nih.gov/pmc/):

(

("nuclear"[All Fields] AND "magnetic"[All Fields] AND "resonance"[All Fields]) OR

("nuclear magnetic resonance"[All Fields]) OR

("nmr"[All Fields])

)

AND

(

("fragment-based"[All Fields] AND "drug discovery") OR

("drug"[All Fields] AND "discovery"[All Fields]) OR

("drug discovery"[All Fields])

)

AND

("2015/1/01"[PubDate] : "2020/12/31"[PubDate])

1.4. Tables

Technique	Common abbreviation	Structural information	Dimensionality	Observed	Labelling	Reference
**Group Selective Saturation Transfer Difference**	GS-STD	Intermolecular interactions between the ^{15}N labelled amino group of the target and the ligand	1D	Ligand	^{15}N	15
**Structural information using Overhauser effects and Selective labelling (SOS) Saturation Transfer Difference in real-time**	SOS-STD	Distance restraints between selectively labelled residues (>3) and the ligand	1D	Ligand	^{2}H	16
**Group Epitope Mapping by Saturation Transfer Difference**	GEM-STD	Quantification of atomic level interactions between the target and the ligand	1D	Ligand	None	17
**Water-Ligand Observed via Gradient Spectroscopy**	WaterLOGSY	Quantification of solvent-exposed protons of the ligand in complex with the target	1D	Ligand	None	18
**Solvent Accessibility, Ligand binding, and Mapping of ligand Orientation by NMR Spectroscopy**	SALMON	Indication of solvent-exposed protons of the ligand in complex with the target	1D	Ligand	None	19
**Target Immobilized NMR Screening**	TINS	Identification of the ligand binding site on the target	1D	Ligand	None	20
**Spin Labels Attached to Protein Side chains as a Tool to Identify interacting Compounds**	SLAPSTIC	Interactions between the target side chain atoms and the ligand	1D	Ligand	Para-magnetic labels	21
**Ligand Proton Pseudocontact Shifts**	PCS	Intermolecular restraints that enable the identification of the target binding site and ligand orientation	Various	Ligand / Target	Para-magnetic labels	22
**Protein-observed ^{19}F NMR**	PrOF	Orthosteric binding site identification	Various	Target	^{19}F	23
**Interligand NOEs for Pharmacophore Mapping**	INPHARMA	Interaction and orientation of two adjacent ligands in a target binding pocket	2D	Ligand	None	24
**Inter-ligand nuclear Overhauser effect**	ILOE	Interaction and orientation of two adjacent ligands in a target binding pocket	2D	Ligand	None	25
**^{19}F chemical exchange saturation transfer (CEST)**	^{19}F-CEST	Structural restraints between a ligand and the target for a complex in intermediate exchange	Various	Target	^{19}F	26
**Nuclear Overhauser Effect**	NOE	Various intra- and inter-molecular distances between ligand atoms and/or ligand-target atoms	Various	Ligand / Target	Various	27–29

Table 1

A list of NMR commonly used techniques for elucidating the structural properties of ligand-target binding events.

1.5. References

1. Chessari, G. and Woodhead, A. J. (2009) From fragment to clinical candidate-a historical perspective. *Drug Discov. Today* **14**, 668–675.
2. Sheng, C. and Zhang, W. (2013) Fragment Informatics and Computational Fragment-Based Drug Design: An Overview and Update. *Med. Res. Rev.* **33**, 554–598.
3. Singh, M., Tam, B. and Akabayov, B. (2018) NMR-fragment based virtual screening: A brief overview. *Molecules* **23**, 233–260.
4. Erlanson, D. A., Fesik, S. W., Hubbard, R. E., Jahnke, W. and Jhoti, H. (2016) Twenty years on: The impact of fragments on drug discovery. *Nat. Rev. Drug Discov.* **15**, 605–619.
5. D. A. Erlanson. http://practicalfragments.blogspot.com Practical Fragments blog.
6. D. A. Erlanson. http://practicalfragments.blogspot.com/2018/10/fragments-in-clinic-2018-edition.html (2018) Fragments in the clinic: 2018 edition.
7. D. A. Erlanson. http://practicalfragments.blogspot.com/2015/01/fragments-in-clinic-2015-edition.html (2015) Fragments in the clinic: 2015 edition.
8. Skinner, S. P. *et al.* (2016) CcpNmr AnalysisAssign: a flexible platform for integrated NMR analysis. *J. Biomol. NMR* **66**, 111–124.
9. O'Boyle, N. M. *et al.* (2011) Open Babel: An Open chemical toolbox. *J. Cheminform.* **3**,.
10. http://openbabel.org The Open Babel Package.
11. McKinney, W. (2011) pandas: a foundational Python library for data analysis and statistics. *Python High Perform. Sci. Comput.* 1–9.
12. Van Der Walt, S., Colbert, S. C. and Varoquaux, G. (2011) The NumPy array: A structure for efficient numerical computation. *Comput. Sci. Eng.* **13**, 22–30.
13. Taschini, S. (2008) Interval Arithmetic: Python Implementation and Applications. *Proc. 7th Python Sci. Conf. (ScyPy 2008).*
14. Bajusz, D., Rácz, A. and Héberger, K. (2015) Why is Tanimoto index an appropriate choice for fingerprint-based similarity calculations? *J. Cheminform.* **7**,.
15. Kövér, K. E., Groves, P., Jiménez-Barbero, J. and Batta, G. (2007) Molecular recognition and screening using a 15N group selective STD NMR method. *J. Am. Chem. Soc.* **129**, 11579–11582.
16. Hajduk, P. J. *et al.* (2004) SOS-NMR: A Saturation Transfer NMR-Based Method for Determining the Structures of Protein−Ligand Complexes. *J. Am. Chem. Soc.* **126**, 2390–2398.
17. Mayer, M. and Meyer, B. (2001) Group epitope mapping by saturation transfer difference NMR to identify segments of a ligand in direct contact with a protein receptor. *J. Am. Chem. Soc.* doi:10.1021/ja0100120.
18. Raingeval, C. *et al.* (2019) 1D NMR WaterLOGSY as an efficient method for fragment-based lead discovery. *J. Enzyme Inhib. Med. Chem.* **34**, 1218–1225.
19. Ludwig, C. *et al.* (2008) SALMON: Solvent accessibility, ligand binding, and mapping of ligand orientation by NMR spectroscopy. *J. Med. Chem.* **51**, 1–3.
20. Vanwetswinkel, S. *et al.* (2005) TINS, target immobilized NMR screening: An efficient and sensitive method for ligand discovery. *Chem. Biol.* **12**, 207–216.
21. Jahnke, W. (2002) Spin labels as a tool to identify and characterize protein-ligand interactions by NMR spectroscopy. *Chembiochem* **3**, 167–73.
22. Xu, D. *et al.* (2018) Ligand Proton Pseudocontact Shifts Determined from Paramagnetic Relaxation Dispersion in the Limit of NMR Intermediate Exchange. *J. Phys. Chem. Lett.* **9**, 3361–3367.

23. Divakaran, A., Kirberger, S. E. and Pomerantz, W. C. K. (2019) SAR by (Protein-Observed) 19F NMR. *Acc. Chem. Res.* **52**, 3407–3418.
24. Sánchez-Pedregal, V. M. *et al.* (2005) The INPHARMA method: Protein-mediated interligand NOEs for pharmacophore mapping. *Angew. Chemie - Int. Ed.* **44**, 4172–4175.
25. Becattini, B. and Pellecchia, M. (2006) SAR by ILOEs: An NMR-based approach to reverse chemical genetics. *Chem. - A Eur. J.* **12**, 2658–2662.
26. Gao, J. *et al.* (2017) Fluorine Pseudocontact Shifts Used for Characterizing the Protein–Ligand Interaction Mode in the Limit of NMR Intermediate Exchange. *Angew. Chemie - Int. Ed.* **56**, 12982–12986.
27. Ni, F. (1994) Recent developments in transferred NOE methods. *Prog. Nucl. Magn. Reson. Spectrosc.* **26**, 517–606.
28. Williamson, M. P. (2018). The transferred NOE. in *Modern Magnetic Resonance* 1–15 doi:10.1007/978-3-319-28388-3_123.
29. Farina, B. *et al.* (2021) A novel approach for studying receptor-ligand interactions on living cells surface by using NUS/T1ρ-NMR methodologies combined with computational techniques: The RGDechi15D-αvβ5 integrin complex. *Comput. Struct. Biotechnol. J.* **19**, 3303–3318.
